# Accelerometry for Gym-Based Training: Comparing Resistance Versus Plyometric Training

**DOI:** 10.3390/s26216681

**Published:** 2026-10-22

**Authors:** Isabel McGillivray, Alistair Murphy, Machar Reid, Thomas Perri, Rob Duffield

**Affiliations:** 1 School of Human Performance, Rehabilitation and Population Health, Faculty of Health, University of Technology Sydney, Sydney, NSW 2021, Australia; 2 Tennis Australia, Melbourne, VIC 3000, Australia; 3 Association of Tennis Professionals, London, England SW19 4DD, UK

**Keywords:** wearable technology, accelerometry, PlayerLoad^TM^, plyometric training, resistance training

## Abstract

This study used inertial measurement units (IMUs) to describe the accelerometry profiles of plyometric and resistance training, compare between sensor placements and gym session types, and evaluate the between-session reliability of accelerometry-derived metrics during plyometric training. Ten high-performance youth tennis players completed two identical plyometric sessions and one resistance session while wearing IMUs at four anatomical locations (thoracic spine, lumbar spine, left ankle, right ankle). Metrics included peak upward acceleration (PUpAcc), 1D upward PlayerLoad™ (1DPL-up), 2D PlayerLoad™ (2DPL), and 3D PlayerLoad™ (3DPL). All metrics were significantly higher at the ankles than the thoracic or lumbar sites (*p* < 0.05), consistent with signal attenuation across the kinetic chain. For vertical loading in isolation, 1DPL-up demonstrated stronger thoracic-ankle relationships (r = 0.47–0.70) although only one of four correlations was statistically significant, compared with PUpAcc (r = −0.67–0.27; all *p* > 0.05). At the thoracic site, between-session reliability during plyometric training was poor for 1DPL-up (ICC = 0.49) and moderate for PUpAcc (ICC = 0.53). All thoracic-derived metrics differentiated plyometric from resistance sessions (*p* < 0.05). These findings indicate that thoracic-mounted IMUs provide moderately reliable, session-sensitive information on peak vertical accelerometry loads in gym-based training. However, the limited inter-sensor agreement for PUpAcc highlights the need for further refinement to enhance practical utility. Overall, accelerometry appears feasible for integrating gym-based training loads into holistic daily training load monitoring.

## 1. Introduction

Modern training load monitoring technologies, including global positioning systems (GPS) and accelerometry, are widely implemented within professional sport for quantifying demands of field- and court-based training [1]. However, despite considerable advances in wearable technology, their application remains largely confined to on-field contexts [2]. Off-field (or off-court) training environments (e.g., gym settings) represent a substantial component of an athlete’s overall training load; however, they are not typically monitored using the same technologies [3]. Training load monitoring practices are consequently fragmented, with different training modalities using disparate, non-comparable technologies and metrics [2,4,5]. This disconnect constrains practitioners’ ability to evaluate cumulative training loads, limiting insight into an athlete’s total training exposure [4]. Indeed, athletes train in systems rather than isolated silos, irrespective of training context or measurement technology [6]. Hence, integrated training load monitoring technologies are needed to capture the full spectrum of training stress, regardless of context (i.e., on- or off-court/field).

Tennis athletes, like those in many high-performance sports, routinely engage in multiple training modalities each day, combining on-court practice with gym-based sessions aimed at developing strength and movement qualities [7]. Plyometric training improves speed and power outcomes, and is often performed in the gym alongside regular resistance training to develop strength [8,9]. These off-court sessions are critical for performance given the extensive physical demands of tennis, including exposures to high ground reaction forces during rapid changes of direction [10,11]. Despite these demands, their training load characteristics remain poorly described within the broader daily training context, especially in relation to how gym-based sessions may complement or contrast with court-based demands [12]. This research gap exists partly because the technologies routinely used for monitoring on-court demands have not yet been validated for use in off-court environments. However, accelerometry is now embedded within standard tennis monitoring systems, with recent advances enabling the reporting of accelerometry-derived on-court metrics, such as stroke counts and movement classifications [13]. Thus, these devices could potentially also quantify gym-based workloads to enable a unified view of daily mechanical stress.

IMUs (inertial measurement units)—often embedded within GPS units—contain accelerometers, gyroscopes and magnetometers that collectively quantify linear accelerations, angular velocities and device orientation across the anteroposterior, mediolateral and vertical axes [14]. Using triaxial accelerometry to capture three-dimensional acceleration allows detailed assessment of multidirectional movement, with PlayerLoad^TM^ quantifying the summed instantaneous rate of change of acceleration across all three axes, providing an indication of cumulative external load [14,15]. Metrics that isolate vertical acceleration, including one-dimensional vertical PlayerLoad^TM^ (1DPL-up), reflecting cumulative vertical loading, and peak upward acceleration (PUpAcc), capturing instantaneous high-intensity impacts, may offer more targeted insight into mechanical stress relating to bone adaptation and injury risk [16]. Vertical forces are a ubiquitous feature of human movement—present in sport-specific actions, plyometrics and resistance exercises—and are closely related to musculoskeletal adaptation, bone health and the risk of bone-related injury and time lost from sport [6,17,18]. This gap presents an opportunity for IMUs to contribute meaningfully to holistic workload monitoring by capturing high-frequency accelerations in the vertical plane, as well as at a three-dimensional level.

A key consideration in applying IMUs for vertical loading analysis is sensor placement. IMU signals measure accelerations local to the segment to which they are attached [19]. This suggests that proximity to the site of impact (i.e., foot or ankle) represents the most biomechanically appropriate location for collecting vertical loading data [6]. In sporting contexts, however, thoracic-mounted sensors are standard, as they capture whole-body motion and are easier to implement consistently [20]. Given the known attenuation of signals as they propagate up the kinetic chain, understanding the relationship between thoracic-mounted IMUs and ankle-derived measures, and the implications of placement for the validity and interpretation of vertical loading metrics in gym-based sessions is essential.

Our previous work [21] provided initial evidence demonstrating that triaxial accelerometry can quantify gym-based movement at the exercise level. Accelerometry-derived metrics reflected the mechanical characteristics of plyometric exercises, showing acceptable reliability and inter-sensor agreement. However, training load monitoring in applied sporting environments is typically conducted at a session level, with practitioners interpreting metrics across the varied exercises and movement patterns that constitute a session in its entirety. It therefore cannot be assumed that the performance and interpretation of accelerometry-derived metrics at the exercise level will translate directly to session-level monitoring. Thus, the next logical step is to extend this investigation to full training sessions and additional session types. Accordingly, this study investigated the ability of IMUs to measure the accelerometry-based training load of gym-based sessions. Specifically, this study aims to (1) describe accelerometry profiles of plyometric and resistance sessions, including metric type and sensor placement; (2) evaluate the reliability of accelerometry-derived metrics during a gym-based plyometric session; and (3) assess the ability of these metrics to discriminate between session types.

## 2. Materials and Methods

### 2.1. Experimental Approach

During a one-week data collection period, high-performance youth athletes completed three standardized gym-based sessions, including two identical plyometric sessions and one resistance session, each scheduled 3–4 days apart and in a fixed order (plyometric one; plyometric two; resistance). These methods were informed by similar work examining plyometric exercises at an individual exercise level [21]. In all sessions, subjects were equipped with four commercial GPS devices (Catapult Vector S7, Catapult Sports, Melbourne, Australia) containing an in-built accelerometer, gyroscope and magnetometer. The devices were oriented so that the Z axis corresponded to the longitudinal axis of the participant’s body and were mounted at the following four anatomical sites: (1) between the scapulae, at the level of the proximal thoracic spine, (2) the lumbar spine at the level of L4/L5, (3) the left ankle, positioned immediately posterior to the medial malleolus, and (4) the right ankle in the equivalent position. The thoracic and lumbar placements were chosen due to their regular use in field and court-based monitoring. For instance, the thoracic spine is a well-validated default GPS location [14,20], while the lumbar spine approximates the body’s center of mass (CoM) and is thus considered the optimal location for capturing whole-body locomotion [22]. Ankle placement followed best-practice recommendations for capturing vertical loading at ground contact [23] in the absence of a true gold-standard measurement tool (e.g., force plates). Gym-based plyometric and resistance sessions were subsequently characterized using accelerometer-derived metrics, including peak upward acceleration, and total 1D upward, 2D and 3D PLs.

### 2.2. Subjects

Ten high-performance youth tennis players (age 16.0 ± 1.6 y, mass 65.2 ± 3.9 kg, stature 171.6 ± 6.72 cm, male n = 6, female n = 4) enrolled in a National Tennis Academy or National Development Program participated in this study. An a priori power analysis (r = 0.50, *p* < 0.05 and power = 0.80) conducted using G*Power (version 3.1.9.6) indicated that 48 exercise-level observations were required. Each participant completed 21 exercise-set observations within each plyometric session (7 exercises × 3 sets) and 27 during the resistance session (9 exercises × 3 sets). However, the present analysis aggregated these observations to the session level, resulting in a subject-level sample of n = 10. The study was therefore underpowered for the current analyses and considered exploratory. Subjects (and guardians if under the age of 18) provided informed written consent (REF NO. ETH24–9549). All subjects were injury-free at the time of testing and refrained from vigorous physical activity in the 24 h prior to participation.

### 2.3. Procedures

Subjects completed gym-based sessions (i.e., two identical plyometric sessions and a resistance session), on three separate occasions, each separated by 3–4 days. Sessions commenced with a 15-min warmup, including a 5-min treadmill run and ~8–10 dynamic exercises. The plyometric session involved three sets of seven exercises, completed in a superset format, as outlined in Table 1. The resistance training session was structured into three blocks (A-C), each comprising three exercises performed in a tri-set sequence for 3 sets, as per Table 2. A 60-s recovery period was provided between all exercises and sets across both sessions. Exercises were manually coded to remove the rest periods from analysis.

Subjects were fitted with four commercial GPS units (Catapult Vector S7, Catapult Sports, Melbourne, Australia), each weighing 102 g and equipped with an in-built accelerometer, gyroscope and magnetometer sampling at 100 Hz. Devices were positioned at 4 standardized anatomical sites as previously outlined: (1) the thoracic spine, housed in the manufacturer-designed harness, (2) attached to the lower spine (approximating the CoM) via physiotherapy tape and (3) the left and (4) right ankles, fixed with physiotherapy tape. To minimize measurement noise, devices were secured as snugly as possible through using appropriately sized vests and firm taping techniques [23].

Following sessions, data were uploaded to the manufacturer’s software (OpenField 3.9, Catapult Sports), where each session was segmented using manual timing notation collected during the session to mark the start and end times. Four key accelerometry-derived metrics were analyzed: (a) peak upward acceleration (PUpAcc), as well as three variants of Catapult Sport’s proprietary PlayerLoad^TM^ metrics–(b) 1D PlayerLoad^TM^ (1DPL-up) in the ‘upward’ (z) axis, (c) 2D PlayerLoad^TM^ (2DPL) in the ‘forwards’ (x) and ‘sideways’ (y) axes and (d) 3D PlayerLoad^TM^ (3DPL) across all axes. Given the significant contribution of movement in the vertical axis to overall loading in field-based sport [24,25], the vertical component was explored in isolation (i.e., PUpAcc). This metric reflects rapid deceleration upon landing, and was defined as the maximum value of the manufacturer-provided *imuAccelerationUp* variable within each segmented session and reported in units of gravitational force (g). According to the manufacturer, this variable represents calibrated acceleration corrected for device tilt relative to the ground [26]; no additional filtering, orientation adjustment or outlier removal was applied by the researchers.

PL^TM^ is a modified vector magnitude developed by Catapult Sports [14] and presented in arbitrary units (AU). It is calculated as the square root of the sum of the squared instantaneous rate of change across three vectors (x, y and z axes) then divided by a scaling factor of 100:

(1) $$\mathrm{PL}=\frac{\sqrt{{\left(a_{x,n}-a_{x,n-1}\right)}^{2}+{\left(a_{y,n}-a_{y,n-1}\right)}^{2}+{\left(a_{z,n}-a_{z,n-1}\right)}^{2}}}{100}$$

1DPL and 2DPL represent the upward and the combined forward/sideways constituents of PL^TM^, respectively, while 3DPL comprises all three axes. As the most commonly used variant across sports, 3DPL demonstrates a 1.9% coefficient of variation (CV) in team sport contexts [14] and validity within 2–5% of calibrated reference values [27]. Additionally, since the duration of the plyometric (10.68 ± 1.58 min) and resistance (15.43 ± 1.43 min) sessions were significantly different (*p* < 0.05), a relative value was utilized for all derivations of PL^TM^ (i.e., PlayerLoad ^TM^ per minute).

### 2.4. Statistical Analyses

Statistical significance was set at *p* < 0.05. Data normality and homogeneity of variance were assessed via the Shapiro-Wilk and Levene test, respectively. All data were log-transformed, as they did not meet the assumptions of normal distribution and/or heteroscedasticity. Means and standard deviations were used to report the instantaneous PUpAcc alongside total values for the three PlayerLoad^TM^ metrics for each session. The first plyometric session was used for analyses addressing aims 1 and 3, while both plyometric sessions were compared for the reliability analysis addressing aim 2.

Regarding the first aim, a one-way ANOVA was performed with post-hoc Tukey’s Honest Significant Difference test to evaluate the effect of sensor placement on each of the metrics. Additionally, across each session type, Pearson’s correlation coefficients were used to assess the relationship of the thoracic and lumbar sensor placements as compared to the best-practice placement at the ankles [23]. Correlation magnitudes were based on previous guidelines [28], with < 0.1: trivial, 0.1–0.29: small, 0.3–0.49: moderate, >0.50: large.

To address the second aim, the consistency of each accelerometry metric from one plyometric session to another was assessed using a two-way mixed-effects model intraclass correlation coefficient—ICC(3,1)—with 95% confidence intervals. Notably, since the duration of the two plyometric sessions (10.68 ± 1.58 min versus 10.62 ± 1.88 min) was not significantly different (*p* > 0.05), the absolute values for all metrics were used in this analysis. ICC was interpreted as <0.5: poor reliability, 0.50–0.74: moderate reliability, 0.75–0.90: good reliability, and >0.90: excellent reliability [29]. Regarding aim three, a one-way ANOVA was conducted with post-hoc Tukey’s Honest Significant Difference to assess the difference between the two session types for all metrics to establish the ability of the metrics to differentiate between the two session types. All analyses were performed in R (RStudio, version 4.4.1, RStudio, Inc.).

## 3. Results

To address aim 1, accelerometry metrics were compared across sensor placements. Table 3 shows the results across the respective plyometric and resistance sessions. All metrics were significantly greater at the respective left and right ankles compared to the thoracic and lumbar spine locations (*p* < 0.05). However, there were no significant differences between the left and right ankle placements (*p* > 0.05). Further, there were no significant differences between the thoracic and lumbar placements for any metric (*p* > 0.05), with the exception of 1DPL-up for the resistance session, whereby significant differences existed between the thoracic and lumbar placements (*p* < 0.05).

To further assess the relationship of sensor placement across each session, Table 4 shows correlation analyses and 95% confidence intervals between sensor locations across the different metrics. Analysis of PUpAcc revealed trivial to large non-significant correlations (r = −0.67–0.27; *p* > 0.05) between the thoracic and ankle sensor placements, as well as small to large non-significant correlations (r = 0.10–0.50; *p* > 0.05) between the lumbar and ankle placements. In contrast, correlations between sensor placement for the PL metrics (i.e., 1DPL-up, 2DPL, 3DPL) were higher across both sessions, including several large, very large and extremely large correlations between the thoracic and lumbar placements as compared to the ankle placements, for all three metrics (e.g., 1DPL-up: r = 0.47–0.92 for 8/8 comparisons; 2DPL: r = 0.55–0.86 for 5/8 comparisons; 3DPL: r = 0.52–0.90 for 6/8 comparisons), and showed significance in varied instances (outlined in Table 4, *p* < 0.05).

**Figure 1:**
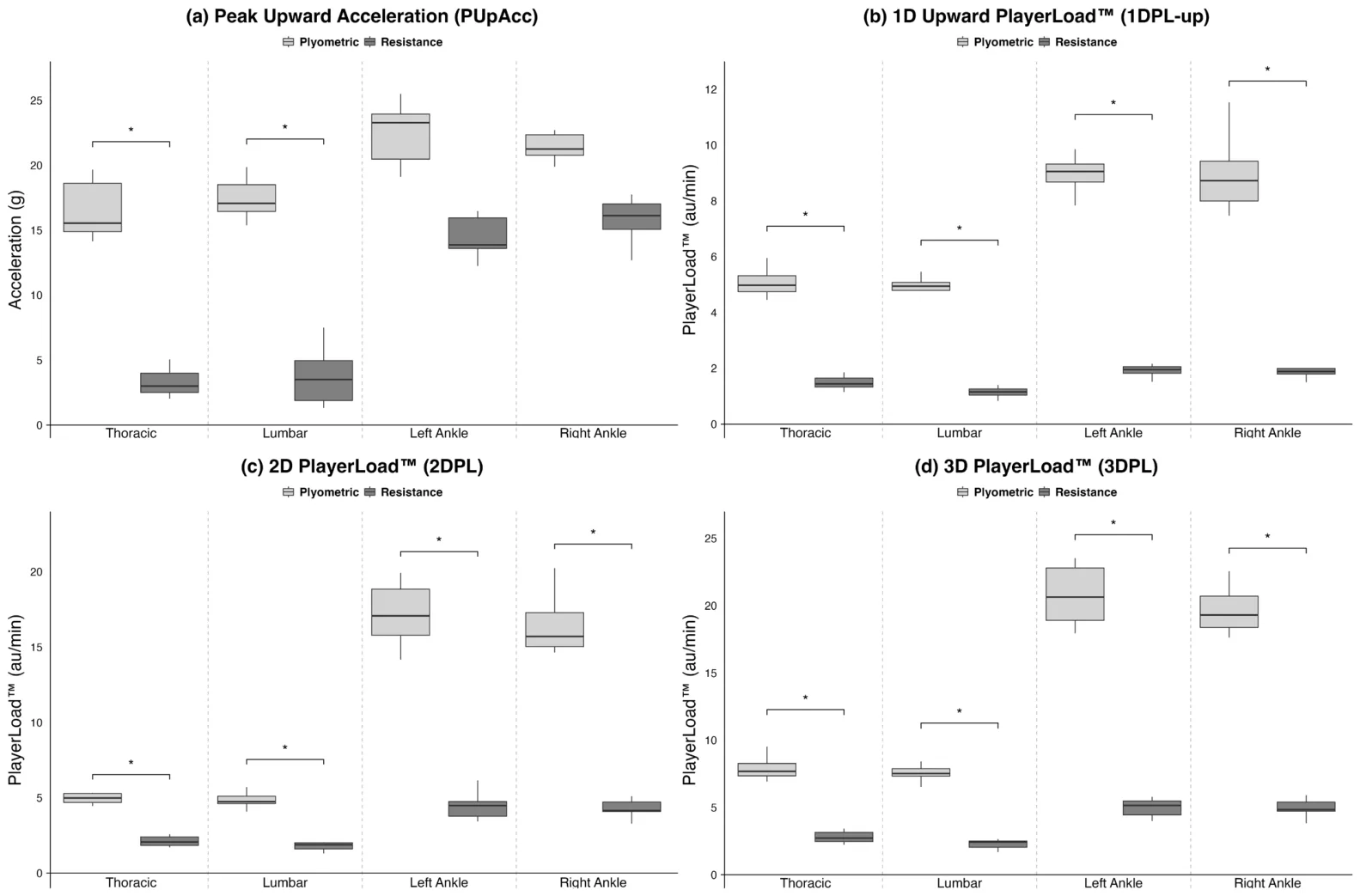
Mean ± SD of peak upward acceleration and 1D, 2D and 3D PlayerLoad^TM^ for each sensor placement across the gym-based plyometric and resistance training sessions. (**a**) Peak Upward Acceleration (PUpAcc); (**b**) 1D Upward PlayerLoad^TM^ (1DPL-up); (**c**) 2D PlayerLoad^TM^ (2DPL); (**d**) 3D PlayerLoad^TM^ (3DPL). * Difference between sessions is statistically significant (i.e., *p* < 0.05).

Regarding the study’s second aim, Table 5 shows the between-session reliability across sensor placements and metrics. Acceptable reliability (ICC > 0.50) was observed for all metrics at the lumbar and right ankle placements, whereas the thoracic spine and left ankle were less consistent (thoracic: 1/4 and left ankle: 2/4). Across metrics, PUpAcc, 2DPL and 3DPL demonstrated the most consistent reliability (3/4 comparisons each), followed by 1DPL-up (2/4). At the thoracic location, PUpAcc and 1DPL-up demonstrated similar between-session reliability estimates (ICC = 0.53 and 0.49, respectively), exceeding that of 2DPL and 3DPL at the same site (ICC ≤ 0.16). To address aim 3, Figure 1 outlines the differences in accelerometry measures by session type. Specifically, within placement, all three PL metrics showed significantly higher values in the plyometric session compared to resistance training (*p* < 0.05). Regarding PUpAcc, significantly higher values were observed in the plyometric session at the thoracic and lumbar locations (*p* < 0.01), but not for the left and right ankle placements (*p* = 0.06 and *p* = 0.26, respectively).

## 4. Discussion

This study utilized triaxial accelerometry to profile gym-based plyometric and resistance training sessions, aiming to (1) characterize accelerometry outputs across session types and sensor placements, (2) assess between-session reliability of key metrics during plyometric training, and (3) determine their ability to differentiate between plyometric and resistance sessions. A consistent attenuation of signal magnitude was observed, with peak acceleration values highest at the ankle and progressively lower at the lumbar and thoracic spine. Of the two vertical loading metrics, cumulative 1DPL-up demonstrated stronger alignment between the ankle and spine-mounted sites than peak measures (PUpAcc), which showed poor associations at the session level. At the thoracic site (the most common placement in applied sport), PUpAcc and 1DPL-up demonstrated similar between-session reliability estimates (ICC = 0.53 and 0.49, respectively), while 2DPL and 3DPL demonstrated poor reliability. Finally, all thoracic-derived metrics successfully distinguished between plyometric and resistance training types.

To our knowledge, this is the first study to characterize session-level accelerometry profiles of plyometric and resistance training by sensor placement and metric type, extending our previous exercise-level work [21]. This extension is practically important because training load is typically interpreted and prescribed at the session level. Evaluating these metrics at this level therefore provides greater insight into their utility within routine athlete monitoring and determines whether findings obtained from individual exercises translate to complete training sessions. As expected, the plyometric session produced greater values across all metrics than the resistance session, with the difference particularly pronounced for the PL metrics that were normalized to session duration. Previous research reports higher mechanical and vertical loading demands of plyometric movements compared to typical resistance training [30], and hence the current accelerometry-based results conform to these previous findings. Specifically, the involvement of altitude landings in plyometric sessions led to the greatest PUpAcc values observed at the ankle. Higher ground-reaction forces reported upon landing from increased drop heights [31] may explain the increased ‘intensity’ and accelerometry profile reported here for plyometric exercises. For practitioners, this application of accelerometry offers promise in describing training load across key gym-based sessions with the same technology used in on-court (or field) training.

Vertical accelerations experienced at foot strike undergo progressive damping as they travel proximally through soft tissue and skeletal structures [20], underpinning the influence of sensor placement on the magnitude of measured loads. This attenuation effect was evident in our data, with spine-mounted IMUs recording significantly lower values than ankle-mounted units across all metrics—aligning with previous observations from tennis-specific actions [32]. Interestingly, all cumulative PL^TM^ values decreased from ankle to thoracic to lumbar for whole-session values, deviating from the anticipated ankle-lumbar-thoracic sequence [22]. This may reflect the contribution of trunk and upper limb motion to accelerations recorded at the thoracic site. Barrett et al. [22] reported that the scapular region exhibits ~6% greater vertical-plane contribution to 3D PlayerLoad^TM^ during treadmill running compared with the center of mass, which is attributed to upper-trunk and shoulder-girdle movement. A similar mechanism may be present in our data, as cumulative thoracic 1DPL-up values may exceed lumbar values due to additional upper-limb and trunk contributions during dynamic exercise and transitional movements. Similar regional differences have been observed in sport-specific contexts involving high trunk contribution [33]. Overall, although thoracic-mounted units may underestimate lower-limb impact magnitudes, the thoracic spine placement remains the standard IMU location within high-performance sport [20], and is well validated in training load monitoring [13]. Accordingly, understanding the pattern of attenuation between the ankles and the thoracic spine is critical, and thoracic-derived values must be interpreted with consideration for both signal damping and the additional contributions of trunk and upper-limb motion when using cumulative acceleration metrics (e.g., PL) to describe a training session.

If vertical-plane accelerometry metrics are to be used as indicators of whole-session bone loading, the most biomechanically appropriate site for capturing vertical impacts is as close as possible to the source of contact (i.e., the feet) [6,20,34]. For PUpAcc, ankle-to-thoracic comparisons across both sessions reveal limited evidence of inter-sensor agreement (r = −0.67–0.27), which is consistent with our previous findings [21], and suggests that the peak thoracic acceleration should not be interpreted as a surrogate measure of ankle-level loading, considering that the ankle is not a criterion measure and may also reflect local segmental kinematics. More broadly, these findings illustrate a conceptual limitation: a single instantaneous value cannot capture the complexity of individual, high-intensity impacts within a set or session. However, PUpAcc may hold applied value as a within-placement marker of high-intensity peaks when integrated within a continuous monitoring paradigm rather than used as a standalone session summary metric. Conversely, 1DPL-up—a cumulative metric—shows higher ankle-to-thoracic correlations (r = 0.47–0.70), reflecting its ability to represent global session loading more effectively; this aligns with findings at the exercise level [21]. 1DPL-up, however, lacks the resolution needed to identify effort-by-effort contributions or peak intensities [35,36], despite the practical importance of understanding such contributions [37]. Collectively, these findings highlight a need for future research on session-level analysis, integrating high-resolution peak data (PUpAcc) into a continuous, zone-based framework. Such work, conceptually similar to heart rate training zones [38], may provide a more nuanced and valid representation of thoracic accelerometry for plyometric load monitoring.

Although the reliability of GPS-derived metrics from commercial IMU devices is generally well-established [39,40,41], there is a lack of research examining accelerometry-derived vertical loading metrics, specifically within gym-based contexts. Surprisingly, the present analysis revealed poor between-session reliability for 1DPL-up (ICC = 0.49), alongside moderate reliability values for PUpAcc (ICC = 0.53); however, their wide, overlapping confidence intervals do not indicate a meaningful difference between the two estimates. These findings contrast markedly with previous vertical PlayerLoad™ reliability values reported from field-based settings, including soccer match play (ICC = 0.99) [25] and treadmill running (ICC = 0.93) [22]. They also diverge from the exercise-level observations in [21], where 1DPL-up typically outperformed PUpAcc (though this did vary by exercise type). With respect to discrimination between session types, all thoracic accelerometry-derived metrics successfully differentiated plyometric from resistance sessions, demonstrating strong sensitivity to differences in exercise accelerometry demands. While no previous studies have directly compared gym-based session types, accelerometry has been shown to discriminate between court/field-based training types (e.g., skill-based, match play, conditioning focus) in sports such as netball [42] and rugby league [43]. Collectively, these findings reinforce the potential for PUpAcc obtained from a thoracic-mounted IMU to monitor and differentiate gym-based loading stimuli in applied settings.

Several limitations warrant consideration when interpreting these findings. Firstly, the small sample size (n = 10) may limit the generalizability of the results and reduce statistical power and precision, potentially obscuring smaller ANOVA effects and increasing uncertainty in the correlation and ICC estimates. Secondly, the absence of a gold-standard comparison tool, such as force plates, constrains our ability to validate accelerometry-derived vertical loading metrics against true ground reaction forces. Third, PUpAcc represents a novel, exploratory metric that captures only a single, instantaneous peak value per session, potentially overlooking important information regarding the distribution and frequency of high-intensity impacts throughout a session. Fourth, natural variability in exercise technique among athletes may contribute to between-session inconsistencies and influence the reliability of accelerometry outputs. Fifth, although sensors were firmly secured to lumbar and ankle sites using standardized fixation procedures, movement or loosening may have introduced measurement error. Finally, while necessary for experimental control, the use of standardized gym-based sessions may not fully represent the variability and complexity inherent to typical training environments, where exercise selection, intensity, volume and execution vary considerably based on individual needs-based programming. These limitations highlight the need for future research employing larger samples, gold-standard validation methods, and protocols that better reflect the dynamic nature of applied high-performance training contexts.

## 5. Conclusions

In summary, this study provides the first session-level comparison of plyometric and resistance training accelerometry profiles across sensor placements and metric types, extending prior exercise-level research to the complete-session level of analysis typically used by practitioners to monitor and prescribe training load. Plyometric sessions elicited substantially greater vertical accelerometry profiles than resistance training. An attenuation effect was evident, reflecting the tendency for trunk-mounted IMUs to underestimate lower-limb loading. Nevertheless, the thoracic location remains the most viable placement for practitioners—demonstrating both sensitivity to session type and moderate between-session reliability for the PUpAcc metric. While thoracic PUpAcc showed limited inter-sensor agreement relative to ankle-derived peak values, this highlights the fundamental limitations of using instantaneous, peak-based metrics to represent session load. The integration of PUpAcc into a continuous, zone-based framework—analogous to heart rate monitoring—may therefore offer a promising approach for improving validity, interpretability and applied utility of thoracic accelerometry in tracking vertical load.

## Figures and Tables

**Table 1:** Plyometric Session.

Exercise	Description	Repetitions
Altitude landings	Stepping off a 42-inch box and absorbing impact using a controlled, soft-landing strategy	3
Countermovement jumps (CMJs)	Rapid eccentric lowering of the center of mass (CoM) immediately followed by a maximal vertical jump with arm swing	6
Stiff drop landings	Stepping off a 12-inch box and performing a controlled, stiff landing with minimal joint flexion	6
Drop jumps	Stepping off a 12-inch box and immediately executing a maximal vertical jump upon landing, minimizing ground contact time	6
Pogo jumps	Quick, low-amplitude repeated jumps with minimal knee bend, targeting ankle stiffness	30
Skater jumps	Lateral bounds performed on alternating legs, landing on one foot and immediately pushing off in the opposite direction	16 (alternating)
Squat Jumps	Starting in ~90° squat position with hands on hips and performing a maximal vertical jump without a countermovement	6

**Table 2:** Resistance Training Session.

Exercise	Description	Repetitions
A: Trap bar deadlift	Controlled hip and knee flexion to lift a hexagonal bar from the floor while maintaining a neutral spine	8
A: 3-point lunge	Lunges performed in three directions (forward, lateral, rotational)	3 each side
A: Half-kneeling landmine press	Barbell press from a stable half-kneeling position with the bar anchored to the floor	8
B: Overhead squat	Full range of motion squat with arms extended overhead holding a dowel	10
B: Romanian deadlift	Hip-hinge movement lowering and raising a barbell to target posterior chain strength	8
B: Bear crawl + bird-dog	Combined lateral crawling with contralateral arm and leg extension	5 each side
C: Back extension GHD	Controlled trunk extension using a glute-ham developer while maintaining spinal and hip alignment	10
C: Pull-ups	Vertical pulling movement with emphasis on spinal and hip alignment	10
C: Seated double-leg calf raise	Slow, controlled ankle plantarflexion on a loaded calf raise machine	10

**Table 3:** Mean ± SD of peak upward acceleration and 1D, 2D and 3D PlayerLoad^TM^ by sensor placement.

Session	Unit Location	(a) Peak Upward Acceleration *(PUpAcc; g)*	(b) Relative 1D Upward PlayerLoad *(1DPL-up; au/min)*	(c) Relative 2D PlayerLoad *(2DPL; au/min)*	(d) Relative 3D PlayerLoad *(3DPL; au/min)*
Plyometric	Thoracic	16.54 ± 2.16 ^b,c^	5.07 ± 0.50 ^b,c^	5.15 ± 0.67 ^b,c^	7.86 ± 0.77 ^b,c^
	Lumbar	17.41 ± 1.52 ^b,c^	4.89 ± 0.51 ^b,c^	4.75 ± 0.57 ^b,c^	7.49 ± 0.83 ^b,c^
	Left Ankle	22.50 ± 2.22	8.98 ± 0.74	17.31 ± 2.15	20.89 ± 2.28
	Right Ankle	21.69 ± 1.72	8.90 ± 1.24	16.43 ± 1.88	20.00 ± 2.30
Resistance	Thoracic	3.72 ± 2.10 ^b,c^	1.48 ± 0.23 ^a,b,c^	2.11 ± 0.33 ^b,c^	2.79 ± 0.43 ^b,c^
	Lumbar	3.70 ± 2.14 ^b,c^	1.19 ± 0.25 ^b,c^	1.86 ± 0.36 ^b,c^	2.37 ± 0.46 ^b,c^
	Left Ankle	14.46 ± 1.53	1.99 ± 0.47	4.46 ± 0.87	5.18 ± 1.05
	Right Ankle	15.90 ± 1.56	1.97 ± 0.37	4.42 ± 0.73	5.13 ± 0.86

^a^ Significant versus Lumbar; ^b^ Significant versus L Ankle; ^c^ Significant versus R Ankle.

**Table 4:** Pearson correlation coefficients and 95% confidence intervals (CI) between sensor placements for peak upward acceleration and 1D, 2D and 3D PlayerLoad^TM^ across the plyometric & resistance sessions.

Relationship		(a) Peak Upward Acceleration *(PUpAcc; g)*		(b) 1D Upward PlayerLoad *(1DPL-up; au/min)*		(c) 2D PlayerLoad *(2DPL; au/min)*		(d) 3D PlayerLoad *(3DPL; au/min)*	
		Correlation	95% CI	Correlation	95% CI	Correlation	95% CI	Correlation	95% CI
**Plyometric**	**T-L**	0.06	−0.591–0.665	0.68 *	0.089–0.917	−0.06	−0.664–0.593	0.38	−0.331–0.814
	**T-LA**	−0.67	−0.933–0.070	0.47	−0.352–0.882	0.07	−0.666–0.739	0.45	−0.378–0.875
	**T-RA**	−0.21	−0.740–0.487	0.53	−0.144–0.871	0.13	−0.542–0.704	0.45	−0.246–0.843
	**L-LA**	0.10	−0.651–0.752	0.57	−0.222–0.910	0.64	−0.112–0.928	0.71 *	0.006–0.942
	**L-RA**	0.18	−0.509–0.726	0.78 *	0.304–0.946	0.80 *	0.342–0.951	0.82 *	0.404–0.957
	**LA-RA**	0.14	−0.625–0.770	0.57	−0.222–0.910	0.88 *	0.454–0.978	0.83 *	0.307–0.969
**Resistance**	**T-L**	0.51	−0.173–0.864	0.74 *	0.198–0.993	0.74 *	0.197–0.933	0.76 *	0.245–0.939
	**T-LA**	0.06	−0.674–0.773	0.70	−0.012–0.940	0.55	−0.249–0.905	0.61	−0.172–0.919
	**T-RA**	0.27	−0.432–0.770	0.63 *	0.008–0.903	0.46	−0.235–0.846	0.52	−0.163–0.866
	**L-LA**	0.50	−0.314–0.891	0.92 *	0.626–0.986	0.86 *	0.380–0.973	0.90 *	0.520–0.981
	**L-RA**	0.18	−0.509–0.726	0.87 *	0.525–0.968	0.81 *	0.368–0.953	0.83 *	0.418–0.959
	**LA-RA**	0.14	−0.628–0.768	0.95 *	0.734–0.991	0.96 *	0.799–0.993	0.96 *	0.803–0.994

<0.1: trivial association, 0.1–0.29: small association, 0.3–0.49: moderate association, 0.5–0.69: large association, 0.7–0.89: very large association and >0.89: extremely large association. * value is statistically significant (i.e., *p* < 0.05). T, thoracic; L, lumbar; LA, left ankle; RA, right ankle.

**Table 5:** Intraclass correlation coefficients (ICC) with 95% confidence intervals.

GPS Location	(a) Peak Upward Acceleration *(PUpAcc; g)*		(b) 1D Upward PlayerLoad *(1DPL-up; au/min)*		(c) 2D PlayerLoad *(2DPL; au/min)*		(d) 3D PlayerLoad *(3DPL; au/min)*	
	ICC	95% CI	ICC	95% CI	ICC	95% CI	ICC	95% CI
**T1 versus T2**	0.53 *	−0.10–0.86	0.49 *	−0.15–0.85	0.00	−0.60–0.60	0.16	−0.49–0.69
**L1 versus L2**	0.72 *	0.21–0.92	0.74 *	0.24–0.93	0.69 *	0.14–0.91	0.72 *	0.20–0.92
**LA1 versus LA2**	0.11	−0.53–0.67	0.36	−0.31–0.79	0.72 *	0.21–0.92	0.61 *	0.02–0.89
**RA1 versus RA2**	0.68 *	0.12–0.91	0.53 *	−0.11–0.86	0.60 *	0.00–0.88	0.56 *	−0.07–0.87

<0.5: poor reliability, 0.50–0.74: moderate reliability, 0.75–0.90: good reliability, >0.90: excellent reliability. * value is statistically significant (i.e., *p* < 0.05). *T1*, thoracic session 1; *T2,* thoracic session 2; *L1,* lumbar session 1; *L2,* lumbar session 2; *LA1*, left ankle session 1; *LA2*, left ankle session 2; *RA1*, right ankle session 1; *RA2*, right ankle session 2.

## Data Availability

The data supporting the findings of this study are available from the corresponding author upon reasonable request. The dataset has not been deposited in a public repository because of the large volume and file size of the high frequency accelerometry data.

## Abbreviations

The following abbreviations are used in this manuscript:

1DPL-up	One-dimensional upward PlayerLoad^TM^
2DPL	Two-dimensional PlayerLoad^TM^
3DPL	Three-dimensional PlayerLoad^TM^
ANOVA	Analysis of variance
AU	Arbitrary units
CI	Confidence interval
CMJ	Countermovement jump
CoM	Centre of mass
CV	Coefficient of variation
GHD	Glute-ham developer
GPS	Global positioning system
ICC	Intraclass correlation coefficient
IMU	Inertial measurement unit
PL	PlayerLoad^TM^
PUpAcc	Peak upward acceleration
SD	Standard deviation
